# Reliability of Pelvic Floor Muscle Assessment with Transabdominal Ultrasound in Young Nulliparous Women

**DOI:** 10.3390/jcm10153449

**Published:** 2021-08-03

**Authors:** Bartosz Zając, Iwona Sulowska-Daszyk, Anna Mika, Artur Stolarczyk, Ewelina Rosłoniec, Aleksandra Królikowska, Marian Rzepko, Łukasz Oleksy

**Affiliations:** 1 Laboratory of Functional Diagnostics, Central Scientific and Research Laboratory, University of Physical Education in Kraków, 31-571 Kraków, Poland; bartosz.zajac@awf.krakow.pl; 2 Institute of Clinical Rehabilitation, University of Physical Education in Kraków, 31-571 Kraków, Poland; anna.mika@awf.krakow.pl (A.M.); ewelinarosloniec@gmail.com (E.R.); 3 Orthopaedic and Rehabilitation Department, Medical University of Warsaw, 02-091 Warszawa, Poland; drstolarczyk@gmail.com (A.S.); loleksy@oleksy-fizjoterapia.pl (Ł.O.); 4 Department of Sports Medicine, Faculty of Health Sciences, Wroclaw Medical University, 50-367 Wrocław, Poland; aleksandra.krolikowska@umed.wroc.pl; 5 Institute of Physical Culture Sciences, Rzeszów University, 35-310 Rzeszów, Poland; mrzepko@ur.edu.pl; 6 Oleksy Medical & Sports Sciences, 37-100 Łańcut, Poland

**Keywords:** pelvic floor muscles, transabdominal ultrasonographic evaluation, reliability, pelvic floor dysfunctions

## Abstract

The aim of this study was to assess the reliability of pelvic floor muscles evaluation via transabdominal ultrasonography in young nulliparous women and to present the methodology for quantitative assessment of the ultrasound image of the pelvic floor muscles visible as displacement of the posterior wall of the bladder, caused by action of the pelvic floor muscles. The study comprised 30 young, Caucasian, nulliparous women (age 22–27; 168.6 ± 5.1 cm; 57.1 ± 11.8 kg) without pelvic floor muscle dysfunctions. The intra-rater, test-retest and inter-rater reliability of pelvic floor muscles evaluation was performed using transabdominal ultrasound at rest and during voluntary contraction. The reliability was assessed at three points of the image (at the middle, on the right and left side). The reliability of the three-point measurement of the pelvic floor muscles transabdominal ultrasound is excellent in the case of intra-rater assessments, both at rest (ICC = 0.98–0.99) and during contraction (ICC = 0.97–0.98); moderate at rest (ICC = 0.54–0.62) and poor during contraction (ICC = 0.22–0.50) in the case of test–retest assessment; excellent at rest (ICC = 0.95–0.96), and good during contraction (ICC = 0.81–0.87) in the case of inter-rater assessment. Transabdominal ultrasound is a reliable method of pelvic floor muscle evaluation. The three-points of assessment used in our study allowed for broader and more comprehensive imaging of the pelvic floor muscle, e.g., for quantitative detection contractility imbalances between the left and right side Due to the fact that understanding mechanisms of pelvic floor muscle functioning is crucial in the therapy of pelvic floor dysfunctions, therefore, reliable, valid tests and instruments are important.

## 1. Introduction

The pelvic floor muscles (PFM) form a deep pelvic diaphragm (m. pubococcygeus, m. puborectalis, m. iliococcygeus), superficial urogenital diaphragm (m. ischiocavernosus, m. bulbospongiosus, m. transversus perinei superficialis), as well as the urethral and anal sphincters [1,2]. The PFMs support the abdomino-pelvic organs, are responsible for bladder continence, trunk stabilization and respiration. They play an important role in generating, maintaining and increasing intra-abdominal pressure for functional tasks [3,4,5,6,7]. Because the bladder is supported by PFM and their fascia, the contraction of the PFMs results in displacement of the bladder base. Chehrehrazi et al. [8] reported transabdominal ultrasound to be a reliable tool for quantifying PFM displacement by means of bladder base movement.

The PFMs may be dysfunctional due to hypo- or hyperactivity. In the first case, the muscles are unable to properly contract, which may lead to urinary and/or fecal incontinence. Contrarily, in the case of hyperactivity, the muscles remain in constant tension or do not relax when physiologically required, e.g., during voiding or defecation. This may lead to constipation, urinary retention, painful bladder syndrome or vestibulodynia [9,10]. Moreover, dysfunctional PFMs are associated with lower back pain [11,12].

The methods commonly used for assessment of PFM functioning in clinical practice are palpation, manometry, dynamometry, electromyography and ultrasound [13,14,15,16,17]. The transabdominal ultrasound is based on the assessment of bladder base movement as an indicator of PFM activity. Using transabdominal ultrasound, it is possible to assess PFM contraction quality and symmetry in both conditions—voluntary and reflex contraction. It is a very practical method, because it does not require exposing intimate parts of the body and can be performed in both men and women of all ages, being quick and easy to use clinically.

It has been shown that the reliability of the bladder base displacement, assessed with transabdominal ultrasound by the same researcher on the same day (measured at only one point located at the middle of the bladder base), ranged between ICC = 0.63–0.94 [15,18,19,20,21]. However, in the measurements performed by different investigators on the same day, the reported reliability was ICC = 0.79–0.94 [21]. Nonetheless, there are no studies in which the test–retest reliability of transabdominal ultrasound imaging of PFM was reported. There is also a lack of research in which the reliability of bladder base displacement at more than one point was evaluated. This approach may be crucial in comprehensive ultrasonographic (USG) evaluation, allowing PFM contraction symmetry assessment.

Due to its practicality, ultrasound imaging of the PFM has become very popular. However, the methodology for quantitative assessment of the ultrasound image has not been reported, therefore its interpretation is still very subjective. Because there is a lack of research in which the reliability of PFM transabdominal ultrasound assessment would be comprehensively evaluated, in this study this is undertaken for the first time.

Thus, the aim of this study was to assess intra-rater, test-retest and inter-rater reliability of the PFM transabdominal ultrasonographic evaluation at rest and during voluntary contraction in young, nulliparous women. This is the first study in which the reliability was assessed at different points on the PFM using comprehensive bladder displacement measurement at three points of the image (at the middle, on the right and left sides). This approach allowed consideration of the existence of PFM asymmetry. Moreover, in this study, a methodology was presented for quantitative assessment of the PFM ultrasound image in detail.

## 2. Materials and Methods

### 2.1. Participants

In this study, 30 young, Caucasian, nulliparous women (age 22–27; 168.6 ± 5.1 cm; 57.1 ± 11.8 kg) without PFM dysfunctions were evaluated. They were recreationally active and did not engage in regular physical training. They did not have any symptoms of urinary incontinence and did not experience any spinal pain in the 6-month period prior to enrolment in the study. They were informed in detail about the research protocol and gave their written informed consent to participate in the study. All procedures were performed in accordance with the 1964 Helsinki declaration and its later amendments. Approval of the Ethical Committee of Rzeszów University (4 January 2015) was obtained for the research. A prospective cross sectional study design was used.

### 2.2. Procedures

**Figure 1:**
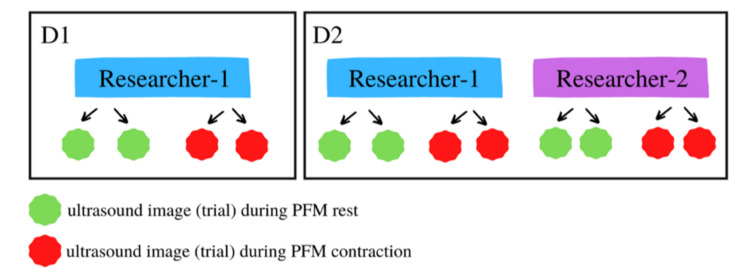
Description of study sequence.

All measurements were performed by two highly experienced researchers, during two days with a 1-day gap between them. Each time, the two measurements of PFM transabdominal ultrasound were performed at rest, while two were carried out during voluntary contraction. The description of the study sequence is shown in Figure 1.

Day 1—On the first day, only researcher-1 performed the measurements.

Day 2—On the second day, the measurements were repeated by researcher-1, and then performed by researcher-2.

The intra-rater reliability was calculated between the two images captured by researcher-1 on Day 1 and independently between the two images captured by researcher-1 on Day 2.

The test–retest reliability was calculated between the measurement from Day 1 and the measurement from Day 2, both performed by researcher-1.

The inter-rater reliability was calculated between the measurement from Day 1 performed by researcher-1 and the measurement from Day 2 performed by researcher-2.

### 2.3. USG of the Pelvic Floor Muscles

Transabdominal ultrasound measurements of PFM function were performed using an ultrasound imaging unit set in B-mode (Honda Electronics CO., LTD., Aichi, Japan, HS-2100V) with a 5 MHz convex transducer. We followed the procedure described by Sherburn et al. [22] and others [15,23,24].

A standardized bladder-filling protocol was used prior to imaging. The evaluated women were asked to fill the bladder by consuming 700–800 mL of water, 1 h before the measurements. This procedure allowed for clear imaging of bladder base movement. The USG measurement was performed in supine position with a pillow underneath the head. The hips and knees were flexed, supported by a pillow under the knees, while the lumbar spine was in a neutral position. The ultrasound probe was placed in transverse orientation, across the midline of the abdomen, immediately superior to the pubic symphysis. The angle of the probe was adjusted to approximately 60° from the vertical position and aimed towards the gluteal or postero-inferior region of the bladder until a clear image of the bladder was visible. Two screens were captured at rest without taking off the probe. The subject was then asked to perform a voluntary PFM contraction; the instructions were‚ “draw in and lift the PFMs”, and then the image was captured at the point of maximal displacement. Next, the women were asked to fully relax, and after 5 s of rest, to contract the PFMs again. Without removing the probe, the second image was captured. The same protocol was repeated for intra-rater, test–retest and inter-rater measurements.

### 2.4. Ultrasound Image Analysis

Analysis of USG measurements was performed by a trained researcher using Image J software (National Institute of Health). Prior to analysis, each image was converted to 8-bit format and scaled. The distance at three points (the middle, right and left sides) of the bladder was measured in millimeters, firstly for the two images from PFM contraction and then the two images at rest. During the analysis the two evaluated images were displayed simultaneously on computer screen one next to other.

**Figure 2:**
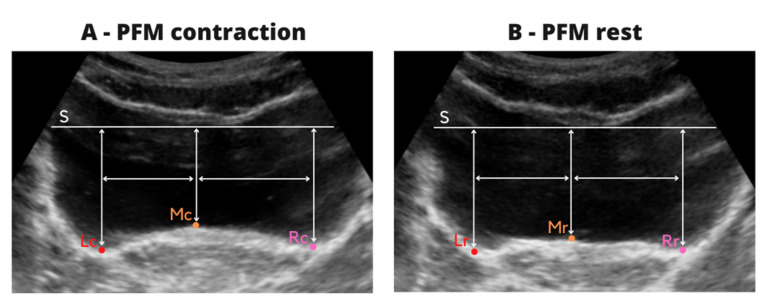
Description of ultrasound image analysis during contraction (**A**) and rest (**B**).

A horizontal tangent (S) was drawn with respect to the lowest point of the bladder apex on both images at the same level. The S line was a reference and then the distance was measured between following points (Figure 2A):

- horizontal tangent (S) and the highest point at the middle of the bladder base (MC),
- horizontal tangent (S) and the lowest point of the bladder base to the left (LC) and right (RC) of the MC point,
- the distance between the MC to LC and MC to RC points was measured and then, the mean value from two images was calculated. This procedure allowed marking of the three points on the bladder base on resting images at the same location, and to calculate the reliability of resting evaluation at the same points as the reliability of contraction.

The same procedure was repeated for the resting images.

A horizontal tangent (S) was drawn on each of the images with respect to the lowest point of the top of the apex of the bladder, and then the distance was measured between the following points (Figure 2B):

- horizontal tangent (S) and the highest point at the middle of the bladder base (MR),
- horizontal tangent (S) and the LR point—located at the base of the bladder to the left of the MR point corresponding to the distance between the points MC–LC on the images from contraction,
- horizontal tangent (S) and the RR point—located at the base of the bladder to the right of the MR point corresponding to the distance between the points MC –RC on the images from contraction.

### 2.5. Statistical Analysis

Statistical analysis was carried out using STATISTICA 12.0 software. To assess the normality of variable distribution, the Shapiro-Wilk test was performed. The intra-rater, test–retest and inter-rater reliability of the variables were determined using Intraclass Correlation Coefficients (ICC). The interpretation of the ICC agreement was performed according to Koo et al. [25]: below 0.50—poor; between 0.50 and 0.75—moderate; between 0.75 and 0.90—good; above 0.90—excellent. The variability within each data set was described using coefficients of variation (CV), based on the mean and SD values. Additionally, Pearson’s linear correlation coefficient (r) was calculated. The two-tailed level of statistical significance was set at *p* < 0.05. Paired *t*-test power analysis determined that at least 25 subjects were required to obtain a power of 0.8 at a two-sided level of 0.05 with the effect size of *d* = 0.8.

## 3. Results

### 3.1. The Intra-Rater Reliability of PFM USG at Rest

The PFM USG resting intra-rater reliability was excellent (ICC = 0.98–0.99). Very strong correlations were noted between measurements (r = 0.97–0.98). All correlations were significant (*p* < 0.05). The coefficient of variation (CV) ranged from 25.4% to 32.8% (Table 1).

### 3.2. The Intra-Rater Reliability of PFM USG during Contraction

The PFM USG intra-rater reliability during contraction was excellent (ICC = 0.97–0.98). Very strong correlations were noted between measurements (r = 0.95–0.97). All correlations were significant (*p* < 0.05). The CV ranged from 22.4% to 34.1% (Table 2).

### 3.3. The Test–Retest Reliability of PFM USG at Rest

The PFM USG test-retest reliability at rest was moderate (ICC = 0.54–0.62). Between measurements, moderate correlations were noted (r = 0.37–0.45). All correlations were significant (*p* < 0.05). The CV ranged from 25.4% to 32.8% (Table 3).

### 3.4. The Test-Retest Reliability of PFM USG during Contraction

PFM USG test–retest reliability during contraction was moderate only for the middle point of measurement in trial 1 (ICC = 0.50). In other measurements, the test–retest reliability was poor (ICC = 0.22–0.44). Also, poor correlations were noted between measurements (r = 0.17–0.33). The correlations were not significant (*p* > 0.05). The CV ranged from 22.4% to 34.1% (Table 4).

### 3.5. The Inter-Rater Reliability of PFM USG at Rest

The PFM USG resting inter-rater reliability was excellent (ICC = 0.95–0.96). Very strong correlations were noted between measurements (r = 0.91–0.93). All correlations were significant (*p* < 0.05). The CV ranged from 21.3% to 30.2% (Table 5).

### 3.6. The Inter-Rater Reliability of PFM USG during Contraction

The PFM USG inter-rater reliability during contraction was good (0.81–0.87 ICC). Between measurements, strong correlations were noted (r = 0.70–0.80). All correlations were significant (*p* < 0.05). The CV ranged from 16.8% to 28.5% (Table 6).

## 4. Discussion

The results of our research showed that the reliability of the 3-point measurement of PFM transabdominal ultrasound is: excellent in the case of intra-rater assessments, both at rest (ICC = 0.98–0.99) and during contraction (ICC = 0.97–0.98); moderate at rest (ICC = 0.54–0.62) and poor during contraction (ICC = 0.22–0.50) in the case of test–retest assessment; excellent at rest (ICC = 0.95–0.96) and good during contraction (ICC = 0.81–0.87) in the case of inter-rater assessment. Due to the fact that understanding PFM functioning is crucial in the therapy of pelvic floor dysfunctions, reliable and valid instruments are especially important. Therefore, in this work, we comprehensively evaluated the reliability of PFM transabdominal ultrasonographic imaging in young nulliparous women as well as at rest during voluntary contraction. Because the assessment of USG image was usually quite subjective, the quantitative analysis suggested in this study may lead to more accurate evaluation of PFM dysfunctions. This may be of clinical significance, which is discussed in further detail below.

It has been previously reported that intra-rater reliability of bladder base displacement during maximal voluntary contraction of PFM, measured by transabdominal ultrasound, and assessed on the image at one point, only ranged between ICC = 0.63–0.94 [15,18,19,20,21]. The inter-rater reliability reported by Murphy et al. [21] was also high (ICC = 0.79–0.94). A good reliability of PFM displacement during voluntary contraction was shown (ICC = 0.93), but was found to be less reliable during reflex contraction (ICC = 0.51) [15].

It should be noted that trans-perineal ultrasonographic imaging of PFM functioning was reported to be more reliable compared to the transabdominal method. In the study of Thompson et al. [15], bladder base displacement measured transperineally was observed to be excellently reliable ICC = 0.91. The authors have suggested that this method of PFM evaluation is more clinically sensitive. Khorasani et al. [20] have reported high (ICC = 0.84), intra-tester reliability of the transabdominal ultrasound measurements assessed in men with chronic prostatitis. Similar findings have been reported by others [15,18,24]. However, in these studies, only intra-rater and/or inter-rater reliability were assessed. In none of the studies was test–retest assessment reported. Moreover, the methodology of USG image assessment used is not clear. In the available research, this procedure was not described in the methods or used only one-point measurement at the middle of the bladder base.

In previous studies, it has been shown that the values of intra-rater and inter-rater reliability were quite similar; nonetheless, some discrepancies between them were also noted. This may be explained by the use of different ultrasound devices (Ultrasonix, Voluson E8, Philips HDI Sono), the use of transducers with varying signal frequencies (from 2 to 8 MHz), insufficient experience of the researchers performing the measurements, or differences in assessment methods of the ultrasound image [19,20].

It should be noted that in our study the ultrasound image assessment was more comprehensive and reliability was calculated separately for the middle, left and right side of the image. The three points of assessment used provided a more comprehensive image of the PFM function. Such an approach may allow for the detection of PFM contractility imbalances between the left and right side. Because the bladder is supported by the PFMs, during contraction, the displacement of the bladder wall should be symmetrical, side-to-side, therefore, the asymmetry of PFM tension due to hypo- or hyperactivity may be diagnosed by ultrasound evaluation [22,23,26]. Thus, using the quantitative evaluation of the image suggested in this study, we may measure the amount of PFM asymmetry at rest as well as asymmetric displacement during contraction. This knowledge may be valuable during the diagnostic process in clinical conditions, where the function of PFM is disrupted. Currently, there are no studies in which the reliability of PFM assessment was reported separately for the left and right sides. Asymmetric work of the PFMs is very common, and to date, this has only been evaluated qualitatively via visual inspection of the USG image, which is highly subjective and vulnerable to errors.

Moreover, this work is the first in which test–retest reliability has been addressed with regard to PFM assessment using transabdominal ultrasound. This issue is of great significance, especially from a clinical perspective, when the PRM ultrasonographic imaging is used for the evaluation of treatment effects. The measurements performed on different days, even by the same highly-experienced researcher, appear more prone to confounding factors (e.g., different amounts of bladder filling), than if they were performed on one day. As was reported in our study, test–retest reliability was only moderate for resting image, and poor for the image taken during PFM contraction. Another important reason for lower reliability is no bony landmark to use as a reference point from which displacement may be measured. This means, that PFM action cannot be measured as an absolute value as in the trans-perineal or intravaginal method, but only as a relative value.

Because PFM dysfunctions are very common, there is a need to create a valid and effective diagnostic method. The three-point assessment of the PFM transabdominal ultrasound appears comparably sensitive to other methods of PFM evaluation, such as dynamometry or electromyography, but much better than manual palpation [8,15]. The dynamometric measurement of the force generated by the PFMs during contraction appeared highly reliable; intra-rater ICC = 0.86–0.96 [27,28,29], as well as inter-rater ICC = 0.86–0.96 [27,30]. However, it was reported that the PFM manual palpation with the use of the modified Oxford Scale presented relatively low diagnostic value, with an inter-rater reliability of 0.33 expressed by the Kappa coefficient [30]. Other authors, based on the Brink Scale, have shown slightly higher inter-rater reliability of manual palpation expressed as Pearson’s correlation coefficient r = 0.44–0.68 [31]. The evaluation of PFM functioning with the use of surface electromyography, measured during maximal voluntary contraction, showed good to excellent intra-rater reliability (ICC = 0.70–0.98), but the test–retest reliability was poor to good (ICC = 0.20–0.76). In another study, the PFM bioelectrical activity was reported as more reliable, and intra-rater, as well as test–retest, showing moderate to excellent reliability of both time-domains and quantitative parameters of PFM recruitment [32]. Nonetheless, as was underlined in those studies, the bioelectrical signal measured in the PFMs at rest and during different types of contraction may be influenced by many factors such as the type of vaginal probe, pelvis position during measurement, contact between the probe and surrounding PFMs, subject age or birth status [14]. Nevertheless, all these methods of PFM evaluation require intravaginal application, which may be a imitating factor. Therefore, transabdominal ultrasound may be easier to apply, especially in specific populations where internal assessment may not be desirable (children, adolescents, victims of sexual abuse, some ethnic groups) [15,24]. This method is also beneficial in case of chronic pelvic conditions, such as deep endometriosis in which internal examination and transvaginal ultrasound performed to study PFM dysfunctions cannot be desirable or painful [33,34]. Assessment of PFM using the intravaginal method may elicit pain, causing pelvic muscle contraction, which can be a confounder. Therefore, transabdominal ultrasound may be beneficial as a non-invasive tool for PFM assessment.

As was shown in this study, the three-point assessment of PMF by transabdominal ultrasound is a reliable method, and therefore, may be used in clinical practice. It is very useful because of its non-invasiveness in the intimate sphere, and allows a lot of information important in the diagnosis of PFM dysfunction, e.g., resting asymmetry or asymmetrical muscle contraction. However, diagnostics with the use of transabdominal ultrasound imaging also have some weaknesses. Bladder base displacement between resting and contraction conditions may be misleading in patients with hypertonic at rest PFM or if these muscles are hypotonic and unable to voluntarily contract. In both cases, the ultrasound image shows lack of movement of the bladder base which may be misleading, and such situations require the use of different assessment methods. Furthermore, movement of the bladder base does not always reflect movement at the bladder neck. Due to the lack of a bony reference point, it may instead sometimes reflect outward movements of the abdominal wall. In obese women or in those with dense abdominal scar tissues, this method of PFM imaging may be difficult to employ [15].

The limitation of this study is the fact that the study group consisted of young, nulliparous women, aged 20–27 years, without PFM dysfunction. Therefore, the USG image quality measured for non-dysfunctional muscles may be higher than in women after childbirth, or in those above the age of 40. Furthermore, the relatively low test–retest reliability observed in our study may be influenced by differences in amount of bladder filling. This may be a problem, especially in patients with reduced functional bladder capacity or bladder urgency. In our study relatively large coefficient of variation for all measurements was also noted. A high value of the coefficient indicates the heterogeneity of the studied population, measuring the dispersion of the variable. Therefore, if PFM both in terms of anthropometric and functional parameters is individually variable, even with strict inclusion/exclusion criteria it is difficult to avoid higher variability within the parameters studied.

## 5. Conclusions

The results of our research have allowed us to indicate that transabdominal ultrasound is a reliable method of PFM evaluation. The three points of assessment used in our study allowed for broader and more comprehensive imaging of the PFM, e.g., for quantitative detection contractility imbalances between the left and right side. Because understanding of the mechanisms of PFM functioning is crucial in therapy of pelvic floor dysfunctions, reliable, valid tests and instruments are important.

## Figures and Tables

**Table 1:** The intra-rater reliability of PFM USG at rest.

Outcome Measure	ICC	r	Mean ± SD (1)	95% CI (1)	CV (%) (1)	Mean ± SD (2)	95% CI (2)	CV (%) (2)
Day 1; Middle (mm)	0.99	0.98 *	4.97 ± 1.6	4.36–5.58	32.8	4.99 ± 1.5	4.41–5.58	31.4
Day 1; Left (mm)	0.99	0.98 *	4.91 ± 1.4	4.36–5.46	29.8	4.90 ± 1.4	4.37–5.44	29.1
Day 1; Right (mm)	0.99	0.98 *	5.29 ± 1.6	4.67–5.91	31.2	5.33 ± 1.5	4.73–5.92	29.9
Day 2; Middle (mm)	0.99	0.98 *	5.83 ± 1.7	5.17–6.49	30.2	5.95 ± 1.6	5.32–6.58	28.3
Day 2; Left (mm)	0.99	0.98 *	5.71 ± 1.5	5.14–6.28	26.7	5.78 ± 1.4	5.23–6.34	25.7
Day 2; Right (mm)	0.98	0.97 *	5.95 ± 1.6	5.32–6.58	28.3	6.06 ± 1.5	5.49–6.64	25.4

ICC—Intraclass Correlation Coefficients; r—Pearson’s correlation coefficient; SD—standard deviation; CV—coefficients of variation; Values are expressed as Mean ± SD; 95% CI—95% Confidence Interval; *—statistical significance (*p* < 0.05); 1—ultrasound image 1; 2—ultrasound image 2.

**Table 2:** The intra-rater reliability of PFM USG during contraction.

Outcome Measure	ICC	r	Mean ± SD (1)	95% CI (1)	CV (%) (1)	Mean ± SD (2)	95% CI (2)	CV (%) (2)
Day 1; Middle (mm)	0.98	0.97 *	5.05 ± 1.5	4.47–5.64	30.9	5.18 ± 1.7	4.52–5.84	34.1
Day 1; Left (mm)	0.97	0.96 *	5.11 ± 1.3	4.61–5.62	26.4	5.22 ± 1.5	4.66–5.79	29.0
Day 1; Right (mm)	0.98	0.97 *	5.52 ± 1.6	4.90–6.13	29.7	5.73 ± 1.8	5.03–6.43	32.7
Day 2; Middle (mm)	0.98	0.97 *	5.87 ± 1.6	5.28–6.47	27.3	5.89 ± 1.6	5.26–6.52	28.5
Day 2; Left (mm)	0.98	0.95 *	5.92 ± 1.3	5.42–6.42	22.9	5.93 ± 1.3	5.43–6.43	22.4
Day 2; Right (mm)	0.98	0.96 *	6.25 ± 1.4	5.71–6.79	23.0	6.28 ± 1.4	5.75–6.81	22.6

ICC—Intraclass Correlation Coefficients; r—Pearson’s correlation coefficient; SD—standard deviation; CV—coefficients of variation; Values are expressed as Mean ± SD; 95% CI—95% Confidence Interval; *—statistical significance (*p* < 0.05) ; 1—ultrasound image 1; 2—ultrasound image 2.

**Table 3:** The test–retest reliability of PFM USG at rest.

Outcome Measure	ICC	r	Mean ± SD (1)	95% CI (1)	CV (%) (1)	Mean ± SD (2)	95% CI (2)	CV (%) (2)
Trial 1; Middle (mm)	0.61	0.44 *	4.97 ± 1.6	4.36–5.58	32.8	5.83 ± 1.7	5.17–6.49	30.2
Trial 1; Left (mm)	0.56	0.39 *	4.91 ± 1.4	4.36–5.46	29.8	5.71 ± 1.5	5.14–6.28	26.7
Trial 1; Right (mm)	0.62	0.45 *	5.29 ± 1.6	4.67–5.91	31.2	5.95 ± 1.6	5.32–6.58	28.3
Trial 2; Middle (mm)	0.61	0.44 *	4.99 ± 1.5	4.41–5.58	31.4	5.95 ± 1.6	5.32–6.58	28.3
Trial 2; Left (mm)	0.54	0.37 *	4.90 ± 1.4	4.37–5.44	29.1	5.78 ± 1.4	5.23–6.34	25.7
Trial 2; Right (mm)	0.58	0.41 *	5.33 ± 1.5	4.73–5.92	29.9	6.06 ± 1.5	5.49–6.64	25.4

ICC—Intraclass Correlation Coefficients; r—Pearson’s correlation coefficient; SD—standard deviation; CV—coefficients of variation; Values are expressed as Mean ± SD; 95% CI—95% Confidence Interval; *—statistical significance (*p* < 0.05) ; 1—ultrasound image 1; 2—ultrasound image 2.

**Table 4:** The test-retest reliability of Pelvic Floor Muscles USG during contraction.

Outcome Measure	ICC	r	Mean ± SD (1)	95% CI (1)	CV (%) (1)	Mean ± SD (2)	95% CI (2)	CV (%) (2)
Trial 1; Middle (mm)	0.50	0.33	5.05 ± 1.5	4.47–5.64	30.9	5.87 ± 1.6	5.28–6.47	27.3
Trial 1; Left (mm)	0.25	0.19	5.11 ± 1.3	4.61–5.62	26.4	5.92 ± 1.3	5.42–6.42	22.9
Trial 1; Right (mm)	0.27	0.21	5.52 ± 1.6	4.90–6.13	29.7	6.25 ± 1.4	5.71–6.79	23.0
Trial 2; Middle (mm)	0.44	0.28	5.18 ± 1.7	4.52–5.84	34.1	5.89 ± 1.6	5.26–6.52	28.5
Trial 2; Left (mm)	0.23	0.19	5.22 ± 1.5	4.66–5.79	29.0	5.93 ± 1.3	5.43–6.43	22.4
Trial 2; Right (mm)	0.22	0.17	5.73 ± 1.8	5.03–6.43	32.7	6.28 ± 1.4	5.75–6.81	22.6

ICC—Intraclass Correlation Coefficients; r—Pearson’s correlation coefficient; SD—standard deviation; CV—coefficients of variation; Values are expressed as Mean ± SD; 95% CI—95% Confidence Interval; 1—ultrasound image 1; 2—ultrasound image 2..

**Table 5:** The inter-rater reliability of PFM USG at rest.

Outcome Measure	ICC	r	Mean ± SD (1)	95% CI (1)	CV (%) (1)	Mean ± SD (2)	95% CI (2)	CV (%) (2)
Trial 1; Middle (mm)	0.96	0.93 *	5.83 ± 1.7	5.17–6.49	30.2	6.07 ± 1.5	5.49–6.64	25.2
Trial 1; Left (mm)	0.95	0.91 *	5.71 ± 1.5	5.14–6.28	26.7	5.99 ± 1.3	5.48–6.50	22.7
Trial 1; Right (mm)	0.95	0.92 *	5.95 ± 1.6	5.32–6.58	28.3	6.18 ± 1.4	5.64–6.72	23.3
Trial 2; Middle (mm)	0.95	0.93 *	5.95 ± 1.6	5.32–6.58	28.3	6.07 ± 1.4	5.54–6.60	23.4
Trial 2; Left (mm)	0.95	0.92 *	5.78 ± 1.4	5.23–6.34	25.7	5.93 ± 1.2	5.46–6.41	21.3
Trial 2; Right (mm)	0.95	0.91 *	6.06 ± 1.5	5.49–6.64	25.4	6.2 ± 1.4	5.66–6.73	23.1

ICC—Intraclass Correlation Coefficients; r—Pearson’s correlation coefficient; SD—standard deviation; CV—coefficients of variation; Values are expressed as Mean ± SD; 95% CI—95% Confidence Interval; *—statistical significance (*p* < 0.05) ; 1—ultrasound image 1; 2—ultrasound image 2.

**Table 6:** The inter-rater reliability of PFM USG during contraction.

Outcome Measure	ICC	r	Mean ± SD (1)	95% CI (1)	CV (%) (1)	Mean ± SD (2)	95% CI (2)	CV (%) (2)
Trial 1; Middle (mm)	0.87	0.78*	5.87 ± 1.6	5.28–6.47	27.3	6.02 ± 1.3	5.68–6.71	22.3
Trial 1; Left (mm)	0.86	0.76*	5.92 ± 1.3	5.42–6.42	22.9	6.26 ± 1.1	5.83–6.70	18.6
Trial 1; Right (mm)	0.82	0.70*	6.25 ± 1.4	5.71–6.79	23.0	6.39 ± 1.4	5.86–6.92	22.0
Trial 2; Middle (mm)	0.87	0.80*	5.89 ± 1.6	5.26–6.52	28.5	6.24 ± 1.3	5.73–6.74	21.6
Trial 2; Left (mm)	0.81	0.71*	5.93 ± 1.3	5.43–6.43	22.4	6.26 ± 1.0	5.86–6.65	16.8
Trial 2; Right (mm)	0.82	0.70*	6.28 ± 1.4	5.75–6.81	22.6	6.49 ± 1.4	5.94–7.04	22.8

ICC—Intraclass Correlation Coefficients; r—Pearson’s correlation coefficient; SD—standard deviation; CV—coefficients of variation; Values are expressed as Mean ± SD; 95% CI—95% Confidence Interval; *—statistical significance (*p* < 0.05) ; 1—ultrasound image 1; 2—ultrasound image 2.

## Data Availability

All data generated or analyzed during this study are included in this published article.
